# Supplementary figures and images for: Lower infant mortality, higher household size, and more access to contraception reduce fertility in low- and middle-income nations
Source: PLoS One. 2023 Feb 22;18(2):e0280260. doi: 10.1371/journal.pone.0280260 (PMC9946217; doi:10.1371/journal.pone.0280260)

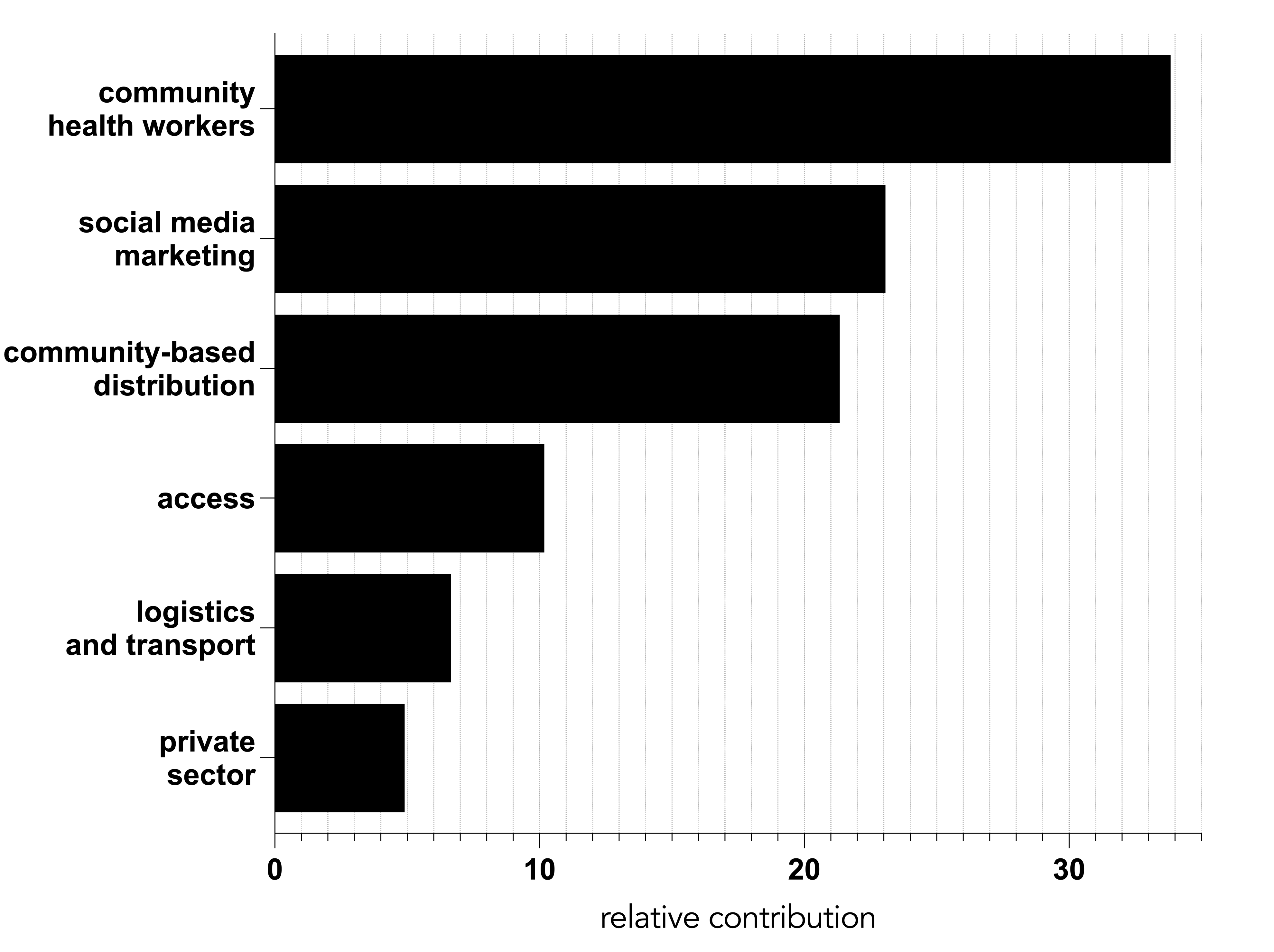

Supplement: S2 Fig — Boosted regression tree results (variable relative performance) for availability of family planning among 52 low- and middle-income countries (available countries in non-imputed dataset). Cross-validation % deviance explained by the final model of 32750 trees: 52.4 ± 7.0%. Community health workers = visitation by a community health worker; social media marketing = social marketing of subsidised contraceptives; community-based distribution = community-based distribution of family-planning; access = ‘access’ index comprising of indicators for availability of family-planning from the Family Planning Effort Index [1]; logistics and transport = logistics and transport; private sector = involvement of private-sector agencies and groups. (TIFF) [file pone.0280260.s002.tiff]

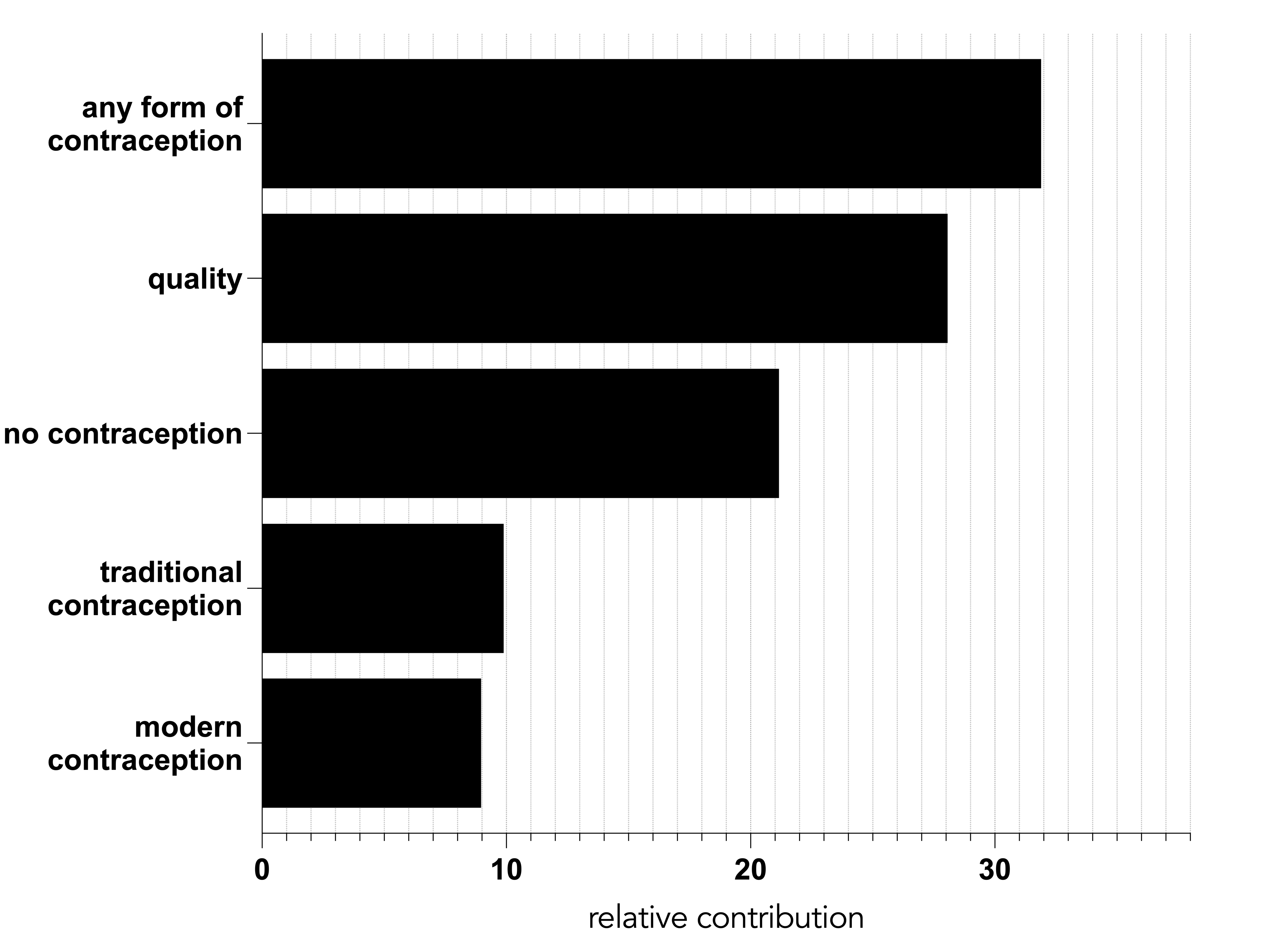

Supplement: S3 Fig — Boosted regression tree results (variable relative performance) for quality of family planning among 56 low- and middle-income countries (available countries in non-imputed dataset). Cross-validation % deviance explained by the final model of 28650 trees: 53.4 ± 10.3%. Any form of contraception = proportion of a population who have access to any form (modern and/or traditional) contraception; quality = ‘quality’ index of family-planning indicators from the National Composite Index on Family Planning [2]; no contraception = proportion of a population who have no access to contraception; traditional contraception = proportion of a population who have access to traditional forms of contraception only; modern contraception = proportion of a population who have access to modern forms of contraception. (TIFF) [file pone.0280260.s003.tiff]

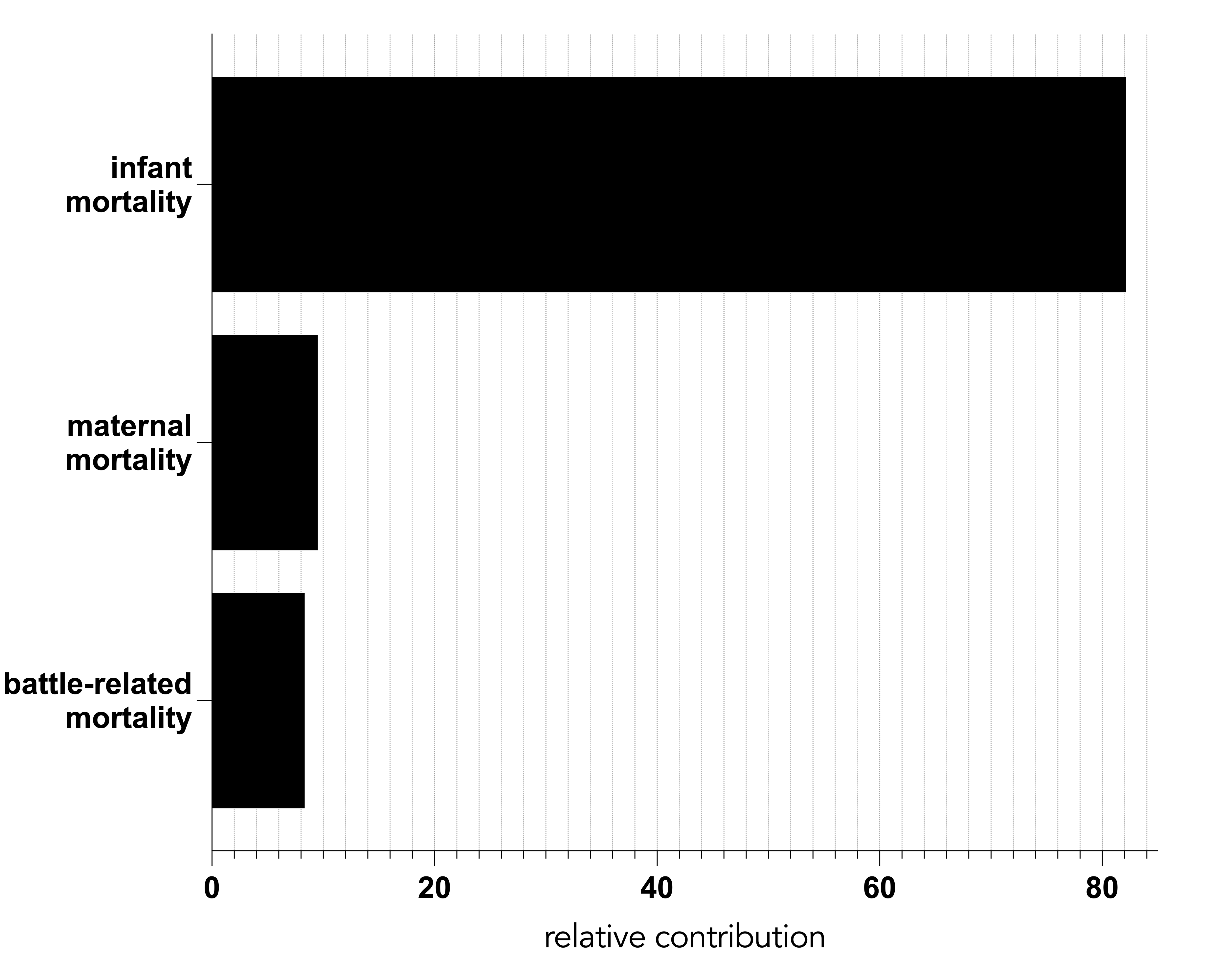

Supplement: S4 Fig — Boosted regression tree results (variable relative performance) for mortality among 29 low- and middle-income countries (available countries in non-imputed dataset). Cross-validation % deviance explained by the final model of 39800 trees: 62.9 ± 14.7%. Infant mortality (deaths per 1000 births); battle-related mortality (deaths per capita); maternal mortality (deaths per 100,000 live births) (TIFF) [file pone.0280260.s004.tiff]

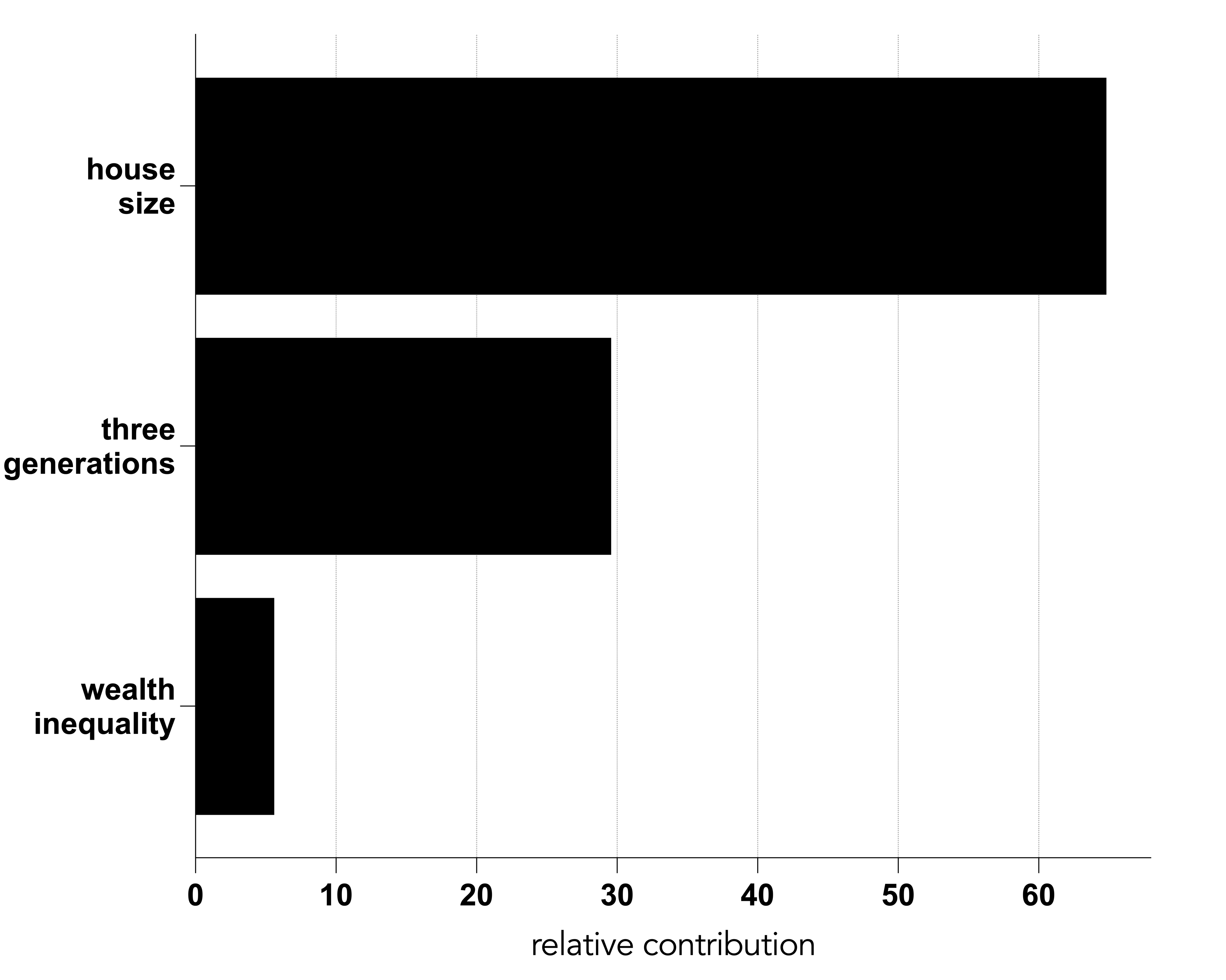

Supplement: S5 Fig — Boosted regression tree results (variable relative performance) for socio-economic indicators among 61 low- and middle-income countries (available countries in non-imputed dataset). Cross-validation % deviance explained by the final model of 39900 trees: 75.3 ± 4.8%. Three generations = households with at least three generations residing; house size = mean number of household members; wealth inequality = bottom 50% of net personal wealth. (TIFF) [file pone.0280260.s005.tiff]

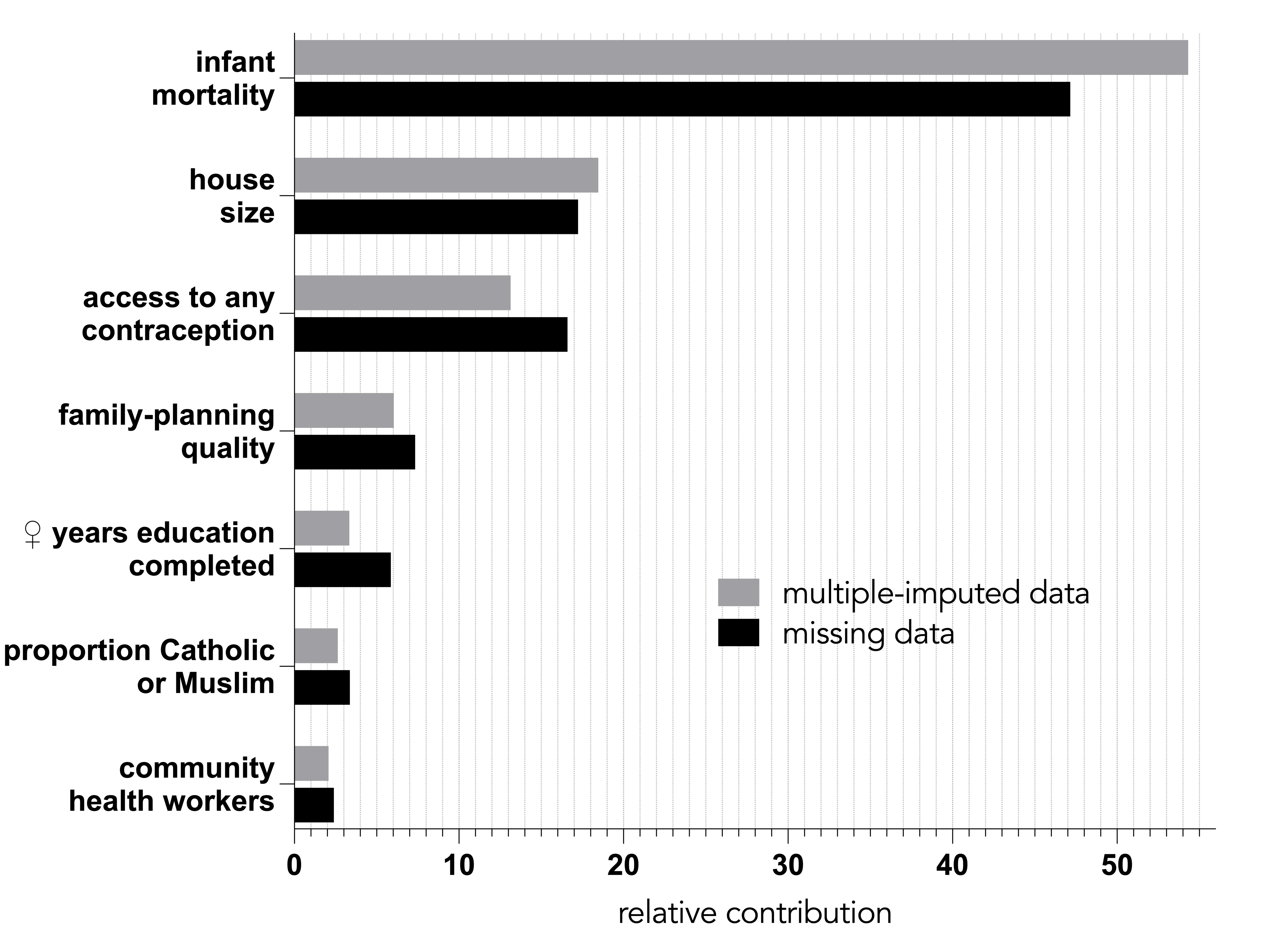

Supplement: S6 Fig — Boosted regression tree results (variable relative performance) of the final phase variables based on multiple-imputed versus missing-data datasets for 64 and 51 low- and middle-income countries, respectively. Cross-validation % deviance explained by the final model: 85.4% ± 3.7% (25100 trees) and 82.6% ± 3.4% (27150 trees) for the imputed and missing datasets, respectively. House size = mean number of individuals per household; community health workers = visitation from community health workers. (TIFF) [file pone.0280260.s006.tiff]
