# Supplementary material for: Lower infant mortality, higher household size, and more access to contraception reduce fertility in low- and middle-income nations
Source: PLoS One. 2023 Feb 22;18(2):e0280260. doi: 10.1371/journal.pone.0280260 (PMC9946217; doi:10.1371/journal.pone.0280260)
Supplement: S1 Table — Correlation (Kendall’s τ) matrix of the highest-ranked variable(s) from each of the six thematic modelling phases (1. family-planning availability, 2. family-planning quality, 3. education, 4. religion, 5. mortality, 6. socio-economics) among 64 low- and middle-income countries. acomm work = visitation by a community healthcare worker; beducation = female years of education; cCathol+Musl = % of population Catholic or Muslim; dinfant mort = infant mortality; eaccess = access to any form of contraception; fquality = quality of family planning index. (DOCX) [file pone.0280260.s007.docx]

|  | comm work^a^ | education^b^ | Cathol+Musl^c^ | infant  mort^d^ | access^e^ | quality^f^ |
| --- | --- | --- | --- | --- | --- | --- |
| education | 0.119 |  |  |  |  |  |
| Cathol+Musl | 0.047 | -0.047 |  |  |  |  |
| infant mort | -0.255 | -0.496 | -0.025 |  |  |  |
| access | 0.109 | 0.403 | -0.146 | -0.443 |  |  |
| quality | 0.115 | -0.009 | -0.117 | -0.023 | -0.059 |  |
| size | -0.092 | -0.520 | 0.330 | 0.389 | -0.516 | 0.009 |
