# Supplementary material for: Lower infant mortality, higher household size, and more access to contraception reduce fertility in low- and middle-income nations
Source: PLoS One. 2023 Feb 22;18(2):e0280260. doi: 10.1371/journal.pone.0280260 (PMC9946217; doi:10.1371/journal.pone.0280260)
Supplement: S2 Table — General linear models for indicators of availability of family-planning in relation to variation in fertility among 52 low- and middle-income countries (available countries in non-imputed dataset). ak = number of parameters; bLL = log-likelihood; cΔBIC = difference in Bayesian information criterion between model and top-ranked model; dwBIC = Bayesian information criterion weight (≈ model probability); e%DE = % deviance explained; fACC = ‘access’ index comprising indicators for availability of family planning from the Family Planning Effort Index [1]; gCBDT = community-based distribution of family planning; hSOCM = social marketing of subsidised contraceptives; iCHW = visitation by a community health worker; jLOGT = logistics and transport; kPRIV = involvement of private-sector agencies and groups; lALL = saturated model (all variables included). (DOCX) [file pone.0280260.s008.docx]

| model | *k^a^* | LL^b^ | ΔBIC^c^ | *w*BIC^d^ | %DE^e^ |
| --- | --- | --- | --- | --- | --- |
| ACC^f^+CBDT^g^ | 3 | -82.06 | 0.00 | 0.23 | 33.2 |
| ACC | 2 | -84.41 | 0.54 | 0.17 | 26.8 |
| ACC+SOCM^h^ | 3 | -82.51 | 0.89 | 0.15 | 32.0 |
| CHW^i^ | 2 | -85.38 | 2.48 | 0.07 | 24.1 |
| ACC+LOGT^j^ | 3 | -83.67 | 3.22 | 0.05 | 28.9 |
| SOCM | 2 | -85.77 | 3.25 | 0.04 | 22.9 |
| PRIV^k^ | 2 | -85.82 | 3.35 | 0.04 | 22.8 |
| SOCM+CHW | 3 | -83.88 | 3.63 | 0.04 | 28.3 |
| ACC+PRIV | 3 | -83.91 | 3.69 | 0.04 | 28.3 |
| LOGT | 2 | -86.06 | 3.84 | 0.03 | 22.1 |
| CBDT | 2 | -86.29 | 4.29 | 0.03 | 21.4 |
| CBDT+CHW | 3 | -84.30 | 4.47 | 0.02 | 27.2 |
| ACC+CHW | 3 | -84.41 | 4.70 | 0.02 | 26.8 |
| SOCM+LOGT | 3 | -84.81 | 5.49 | 0.01 | 25.7 |
| PRIV+CHW | 3 | -84.88 | 5.64 | 0.01 | 25.5 |
| CHW+LOGT | 3 | -85.35 | 6.57 | 0.01 | 24.2 |
| CBDT+SOCM | 3 | -85.40 | 6.67 | 0.01 | 24.0 |
| PRIV+LOGT | 3 | -85.56 | 6.99 | 0.01 | 23.6 |
| PRIV+SOCM | 3 | -85.57 | 7.02 | 0.01 | 23.5 |
| ACC+CBDT+SOCM+LOGT | 5 | -81.57 | 7.32 | 0.01 | 34.4 |
| PRIV+CBDT | 3 | -85.81 | 7.49 | 0.01 | 22.8 |
| CBDT+LOGT | 3 | -85.85 | 7.57 | 0.01 | 22.7 |
| ACC+CBDT+SOCM+CHW+LOGT | 6 | -80.78 | 9.91 | <0.01 | 36.4 |
| ALL^l^ | 7 | -80.71 | 13.94 | <0.01 | 36.6 |
| *intercept-only* | 1 | -107.25 | 42.06 | <0.01 | - |
