# Supplementary material for: Lower infant mortality, higher household size, and more access to contraception reduce fertility in low- and middle-income nations
Source: PLoS One. 2023 Feb 22;18(2):e0280260. doi: 10.1371/journal.pone.0280260 (PMC9946217; doi:10.1371/journal.pone.0280260)
Supplement: S3 Table — General linear models for indicators of quality of family-planning in relation to variation in fertility among 56 low- and middle-income countries (available countries in non-imputed dataset). ak = number of parameters; bLL = log-likelihood; cΔBIC = difference in Bayesian information criterion between model and top-ranked model; dwBIC = Bayesian information criterion weight (≈ model probability); e%DE = % deviance explained; fQUA = ‘quality’ index from the National Composite Index of Family Planning [2]; gNC = access to no form of contraception; hAC = access to any form of contraception; iMC = access to modern contraceptives; kTC = access to traditional contraceptives. (DOCX) [file pone.0280260.s009.docx]

| model | *k^a^* | LL^b^ | ΔBIC^c^ | *w*BIC^d^ | %DE^e^ |
| --- | --- | --- | --- | --- | --- |
| QUA^f^+NC^g^ | 3 | -67.79 | 0.00 | 0.50 | 65.5 |
| QUA+AC^h^ | 3 | -67.80 | 0.01 | 0.50 | 65.5 |
| QUA+MC^i^ | 3 | -72.92 | 10.25 | <0.01 | 58.6 |
| QUA+AC+MC+TC^k^+NC | 6 | -67.19 | 11.27 | <0.01 | 66.2 |
| MC+NC | 3 | -80.86 | 26.14 | <0.01 | 52.7 |
| AC+MC | 3 | -80.87 | 26.15 | <0.01 | 52.7 |
| NC | 2 | -83.48 | 27.22 | <0.01 | 48.4 |
| AC | 2 | -83.48 | 27.22 | <0.01 | 48.4 |
| TC+NC | 3 | -81.95 | 28.32 | <0.01 | 51.0 |
| AC+TC | 3 | -81.96 | 28.33 | <0.01 | 51.0 |
| AC+NC | 3 | -83.41 | 31.24 | <0.01 | 48.6 |
| MC+TC | 3 | -87.23 | 38.87 | <0.01 | 41.7 |
| QUA+TC | 3 | -89.19 | 42.79 | <0.01 | 25.9 |
| MC | 2 | -94.68 | 49.61 | <0.01 | 25.6 |
| QUA | 2 | -95.05 | 50.35 | <0.01 | 15.9 |
| TC | 2 | -98.29 | 56.84 | <0.01 | 16.2 |
| *intercept-only* | 1 | -107.25 | 70.60 | <0.01 | - |
