# Supplementary material for: Lower infant mortality, higher household size, and more access to contraception reduce fertility in low- and middle-income nations
Source: PLoS One. 2023 Feb 22;18(2):e0280260. doi: 10.1371/journal.pone.0280260 (PMC9946217; doi:10.1371/journal.pone.0280260)
Supplement: S4 Table — General linear models for indicators of mortality in relation to fertility among 29 low- and middle-income countries (available countries in non-imputed dataset). ak = number of parameters; bLL = log-likelihood; cΔBIC = difference in Bayesian information criterion between model and top-ranked model; dwBIC = Bayesian information criterion weight (≈ model probability); e%DE = % deviance explained; fIM = infant mortality (deaths per 1000 births); gBRD = battle-related deaths per capita; hMM = maternal mortality (deaths per 100,000 live births). (DOCX) [file pone.0280260.s010.docx]

| Model | *k^a^* | LL^b^ | ΔBIC^c^ | *w*BIC^d^ | %DE^e^ |
| --- | --- | --- | --- | --- | --- |
| IM^f^+BRD^g^ | 3 | -29.74 | 0.00 | 0.52 | 87.7 |
| IM+MM^h^+BRD | 4 | -28.42 | 1.52 | 0.24 | 88.7 |
| IM+BRD+IM*BRD | 4 | -29.14 | 2.96 | 0.12 | 88.2 |
| IM+MM+BRD+IM*BRD | 5 | -27.74 | 4.32 | 0.06 | 89.3 |
| IM+MM+BRD+IM*MM | 5 | -28.12 | 5.08 | 0.04 | 89.0 |
| IM+MM+BRD+IM*BRD+IM*MM | 6 | -27.31 | 7.62 | 0.01 | 89.6 |
| BRD | 2 | -44.56 | 25.47 | <0.01 | 65.7 |
| MM+BRD | 3 | -44.55 | 29.61 | <0.01 | 65.7 |
| IM | 2 | -85.23 | 106.83 | <0.01 | 49.7 |
| IM+MM | 3 | -85.02 | 110.56 | <0.01 | 50.1 |
| IM+MM+IM*MM | 4 | -83.52 | 111.72 | <0.01 | 52.4 |
| *intercept-only* | 1 | -107.25 | 146.70 | <0.01 | 0.0 |
| MM | 2 | -107.25 | 150.86 | <0.01 | - |
