# Supplementary material for: Lower infant mortality, higher household size, and more access to contraception reduce fertility in low- and middle-income nations
Source: PLoS One. 2023 Feb 22;18(2):e0280260. doi: 10.1371/journal.pone.0280260 (PMC9946217; doi:10.1371/journal.pone.0280260)
Supplement: S5 Table — General linear models for indicators of socio-economics in relation to fertility among 61 low- and middle-income countries (available countries in non-imputed dataset). ak = number of parameters; bLL = log-likelihood; cΔBIC = difference in Bayesian information criterion between model and top-ranked model; dwBIC = Bayesian information criterion weight (≈ model probability); e%DE = % deviance explained; fGEN = proportion of household with three generations residing; gHS = household size (number of members); hWLTH = bottom 50% of net personal wealth from the World Inequality Database. (DOCX) [file pone.0280260.s011.docx]

| model | *k^a^* | LL^b^ | ΔBIC^c^ | *w*BIC^d^ | %DE^e^ |
| --- | --- | --- | --- | --- | --- |
| GEN^f^+HS^g^ | 3 | -72.66 | 0.00 | 0.74 | 63.9 |
| WLTH^h^+GEN+HS | 4 | -71.61 | 2.07 | 0.26 | 65.1 |
| WLTH+HS | 3 | -86.51 | 27.70 | <0.01 | 43.1 |
| HS | 2 | -90.19 | 30.91 | <0.01 | 35.8 |
| WLTH+GEN | 2 | -101.21 | 52.94 | <0.01 | 7.8 |
| *intercept-only* | 2 | -106.44 | 63.41 | <0.01 | 2.5 |
| WLTH | 3 | -72.66 | 0.00 | <0.01 | 63.9 |
