# Supplementary material for: Lower infant mortality, higher household size, and more access to contraception reduce fertility in low- and middle-income nations
Source: PLoS One. 2023 Feb 22;18(2):e0280260. doi: 10.1371/journal.pone.0280260 (PMC9946217; doi:10.1371/journal.pone.0280260)
Supplement: S1 File — (DOCX) [file pone.0280260.s012.docx]

Availability of family planning is based on the ‘access’ index in the Family Planning Effort database [1]: (*a*) access to the intrauterine device, (*b*) access to contraceptive pills, (*c*) access to injectables, (*d*) access to female sterilisation, (*e*) access to male sterilisation, (*f*) access to condoms, (*g*) access to implants, (*h*) access to emergency contraception, (*i*) access to safe abortion, (*j*) sterilisation permanence explained by a trained healthcare worker, (*k*) access to intrauterine device removal, and (*l*) access to implant removal. Additional indices from the Family Planning Effort database [1] include : (*a*) involvement of private-sector agencies and groups, (*b*) community-based distribution, (*c*) social marketing (extent of coverage by a social marketing program that subsidise contraceptive sales), (*d*) community health workers (extent of population visited by healthcare workers to educate about family planning and maternal and child health), and (*e*) logistics and transport (extent to which logistics and transport networks are sufficient in maintaining contraceptive supplies and equipment at all services, time and levels).

The quality of the family planning index from the National Composite Index on Family Planning [2] includes: (*a*) standard of practices in line with the World Health Organization, (*b*) guidelines on task-sharing, (*c*) indicators used by public family planning services, (*d*) indicators used by private family planning services, (*e*) structures in place to address quality, (*f*) information collected regarding informed choice and provider bias, (*g*) training programs for workers, (*h*) logistics and transport sufficient to supply contraceptives and related equipment at all times (*i*) adequate supervision and monitoring in place, (*j*) sterilisation permanence education to clients, (*k*) proportion of population who have access to intrauterine device removal, (*l*) proportion of population who have access to implant removal. We used the data from 2017, but if unavailable, we used the 2014 data instead (for Benin, Indonesia, South Africa, Turkey, and Yemen). Additional indices from the Demographic and Health Surveys [3] and Multiple Indicator Cluster Surveys [4] include: (*a*) percentage of the population using any form of contraception, (*b*) percentage of the population using a modern contraceptive method, (*c*) percentage of the population using a traditional contraceptive method, and (*d*) percentage of a population using no form of contraceptive method.

Female education was obtained from the Demographic and Health Surveys and Multiple Indicator Cluster Surveys [3] as weighted mean number of years of completed education for women aged 15-49 years.

Religion data was obtained from the Association of Religion Data Archives (thearda.com). The percentage of the population adhering to either Catholicism or Islam were combined.

Infant and maternal mortality data were from the 2020 World Health Organization Global Health Observatory data repository [5], as well as the average number of battle-related deaths (2015–2020).

To account for variation in socio-economic conditions among countries, we obtained the following variables: (*i*) net personal wealth of the bottom 50% of the population (*p0p50*) from the World Inequality Database [6], (*ii*) mean number of household members from the Demographic and Health Surveys and Multiple Indicator Cluster Surveys (statcompiler.com), and (*iii*) percentage of households with three generations residing from the Demographic and Health Surveys and Multiple Indicator Cluster Surveys (statcompiler.com).

**References**

**1.** track20.org/pages/data_analysis/policy/FPE.php.

**2.** track20.org/pages/data_analysis/policy/NCIFP.php.

**3.** dhsprogram.com.

**4.** mics.unicef.org.

**5.** data.worldbank.org.

**6.** wid.world/data.
